# Prognostic value of glucose-to-lymphocyte ratio for all-cause mortality and consciousness impairment in critically ill cerebrovascular disease patients

**DOI:** 10.1186/s40001-025-03378-6

**Published:** 2025-11-28

**Authors:** Yan Li, Lei Yu, Miaomiao Fu, Lin Liu, Fengyue Han, Yue Qiao, Binglong Li, Xia Gao

**Affiliations:** 1 Department of Neurology, Jinan Third People’s Hospital, No. 1, Wangsheren North Street, Licheng District, Jinan, 250100 China; 2 Department of Cardiology, Jinan Third People’s Hospital, Jinan, China; 3 Physical Examination Center, Jinan Third People’s Hospital, Jinan, China; 4 Department of Neurology, Xuanwu Hospital, Capital Medical University, Beijing, China; 5 Department of Medical Administration, Jinan Third People’s Hospital, Jinan, China

**Keywords:** Glucose-to-lymphocyte ratio, Cerebral infarction, Non-traumatic cerebral hemorrhage, Intensive care unit, Mortality, MIMIC database

## Abstract

**Background:**

The glucose-to-lymphocyte ratio (GLR) is a promising biomarker linked to metabolic status and inflammation, with prognostic value in various diseases. However, its role in predicting outcomes for patients with cerebrovascular diseases, particularly in intensive care units, remains unclear.

**Methods:**

Data from 1,810 intracerebral hemorrhage (ICH) and 2060 ischemic stroke patients in the Medical Information Mart for Intensive Care IV database were analyzed. Cox proportional hazards models were used to evaluate the relationship between GLR and in-hospital mortality, while logistic regression assessed its association with consciousness impairment. Restricted cubic spline analysis was performed to examine potential non-linear links. The incremental predictive value of GLR was evaluated using decision curve analysis and integrated discrimination improvement indices.

**Results:**

Elevated GLR was independently associated with higher in-hospital mortality in both ICH (hazard ratio [HR]: 1.016, *P* = 0.004) and ischemic stroke patients (HR: 1.013, *P* = 0.009). It was also linked to increased risk of consciousness impairment in ICH (odds ratio [OR]: 1.014, *P* = 0.043) and ischemic stroke (OR: 1.015, *P* = 0.013). Non-linear relationships between GLR and mortality were observed in ICH patients (*P* for non-linearity < 0.001). Incorporating GLR into existing clinical scoring systems improved their predictive power (all *P* < 0.05).

**Conclusion:**

GLR is a valuable predictor of severe consciousness impairment and in-hospital mortality in intensive care unit (ICU) patients with cerebrovascular diseases.

**Supplementary Information:**

The online version contains supplementary material available at 10.1186/s40001-025-03378-6.

## Background

Cerebrovascular diseases remain a significant cause of mortality and long-term disability worldwide [1]. Despite remarkable progress in medical management, cerebrovascular diseases remain a major global health challenge, with large artery atherosclerotic and cardioembolic stroke subtypes having particularly poor short-term prognosis [2]. Glycemic status serves as a critical determinant in the pathogenesis, progression, and clinical outcomes of cerebrovascular diseases [3]. Recent studies have shown that elevated blood glucose levels are associated with higher mortality among critically ill patients and may significantly impact the clinical outcomes of cerebrovascular diseases [4]. Hyperglycemia has the potential to exacerbate cerebrovascular damage and enlarge the infarct size by inducing oxidative stress, damaging the blood–brain barrier, and enhancing vasoconstriction [5]. Furthermore, glycemic fluctuations are not only linked to acute neuronal injury but also adversely affect long-term recovery, recurrence rates, and mortality risk. The correlation between the immune system and cerebrovascular diseases has attracted increasing attention. Lymphocytes, being pivotal elements of the immune response, serve as established biomarkers for systemic inflammation and disease progression in various pathological conditions [6]. Studies have shown that low lymphocyte counts are associated with poor outcomes and increased all-cause mortality in patients with cerebrovascular diseases [7].

The glucose-to-lymphocyte ratio (GLR), a recently introduced composite biomarker, offers a unique representation of an individual's metabolic and immune status. By integrating two accessible clinical parameters––blood glucose and lymphocyte count––GLR emerges as a valuable marker for evaluating disease severity and predicting clinical outcomes. Elevated GLR has been demonstrated as a significant prognostic predictor in various conditions, including multiple malignancies and severe respiratory diseases [8–13]. In cerebrovascular diseases, GLR may reflect both metabolic disturbances and inflammatory responses, suggesting its potential as a prognostic marker. Despite numerous studies exploring the prognostic significance of blood glucose levels and lymphocyte counts separately in cerebrovascular diseases [14, 15], evidence regarding the prognostic value of GLR in cerebrovascular diseases remains limited.

The aim of this study is to evaluate the association of GLR with severe consciousness impairment and mortality in patients with cerebrovascular diseases through comprehensive analyses, including its potential integration into existing clinical prediction models.

## Method

### Study population

The data for this study were extracted from the MIMIC-IV (Medical Information Mart for Intensive Care IV) database, version 2.2, a single-center, longitudinal cohort spanning from 2008 to 2019 [16]. Data extraction was conducted by one of the authors, Yue Qiao, who met the requirements for accessing the database (ID: 13512319). This study followed the guidelines outlined by the Strengthening the Reporting of Observational Studies in Epidemiology (STROBE) [17].

Patients were included if they had a diagnosis of non-traumatic cerebral hemorrhage or cerebral infarction, identified according to the International Classification of Diseases, Ninth Revision (ICD-9), or Tenth Revision (ICD-10). The exclusion criteria were as follows: (1) patients who were not admitted to the ICU; (2) follow-up time less than 24 h; (3) patients lacking valid glucose and lymphocyte data; and (4) patients with multiple admissions, only the first admission was included in the analysis.

### Data collection

We used Structured Query Language (SQL) with PostgreSQL to extract data from the MIMIC-IV database, including demographic details, physiological measurements, laboratory measurements, comorbidities, in-hospital mortality, and relevant clinical scores. The extraction of potential variables was as follows: (1) demographic information including age, gender, and race; (2) physiological measurements, which comprised systolic blood pressure (SBP), diastolic blood pressure (DBP), heart rate, and respiratory rate; (3) laboratory measurements including glucose, lymphocytes, alanine aminotransferase (ALT), aspartate aminotransferase (AST), international normalized ratio (INR), partial thromboplastin time (PTT), hemoglobin, platelets, potassium, sodium, and white blood cells (WBC); (4) comorbidities and personal history of patients were identified based on ICD-9 and ICD-10, including anemia, malignancy, chronic kidney disease (CKD), hyperlipidemia, diabetes, hypertension, long-term use of antiplatelet agents/anticoagulants, alcohol abuse, and tobacco use; and (5) scores of clinical severity include Glasgow coma scale (GCS) score, acute physiology score III (APSIII), oxford acute severity of illness score (OASIS), and simplified acute physiology score (SAPSII).

The follow-up period began upon admission and continued until either death or discharge. The GLR index was obtained as the [fasting blood glucose level (mmol/L) /serum lymphocyte (10^9 cells/L)] on the first day of admission. To minimize reverse causation bias, any data collected after the occurrence of outcome events were excluded from the analysis. In the MIMIC-IV database, to avoid potential bias, variables with less than 20% missing data were imputed using a random forest algorithm (trained using other non-missing variables). If a variable had more than 20% missing data, it was excluded from the analysis [18, 19].

### Outcome measures

The primary outcome was in-hospital all-cause mortality, and the second outcome was the occurrence of severe impairment of consciousness (defined as a GCS score ≤ 8 within 30 days after admission).

### Statistical analysis

Since the data exhibited a non-normal distribution, continuous variables were presented as median (interquartile range [IQR]), and group comparisons were performed using the Mann–Whitney U test. Categorical variables are expressed as frequencies and percentages, with differences assessed using the Chi-square test or Fisher's exact test. An optimal GLR cutoff value was determined using maximally selected rank statistics, and patients were subsequently categorized into high and low GLR groups. Kaplan–Meier survival analysis was performed to evaluate survival rates between different GLR level groups, with differences assessed by log-rank test. The primary outcome, in-hospital mortality, was assessed using Cox proportional hazards models to evaluate the association between GLR and mortality, expressed as hazard ratios (HRs) with 95% confidence intervals (CIs). Binary logistic regression was applied to evaluate the relationship between GLR and the secondary outcome, severe consciousness disturbance (defined as a GCS score ≤ 8 within 30 days of admission), expressed as odds ratios (ORs) with 95% CIs. The relationship between GLR and outcomes was assessed through three models: Model 1 was unadjusted; Model 2 was adjusted for demographic characteristics (age, gender, and race); and Model 3 was fully adjusted for potential confounders including demographics, comorbidities (alcohol use, anemia, cardiovascular disease, chronic kidney disease, diabetes, malignancy, hyperlipidemia, hypertension, and tobacco use), and clinical parameters (ALT, AST, hemoglobin, INR, platelet count, potassium, PTT, systolic blood pressure, sodium, temperature, and white blood cell count). The potential non-linear relationship between GLR and in-hospital mortality was explored using restricted cubic splines (RCS). The predictive performance of GLR, both as a continuous and categorical variable, was assessed by the area under the receiver operating characteristic curve (AUC). Clinical decision curve analysis (DCA) was performed, and the integrated discrimination improvement (IDI) was calculated to evaluate the incremental predictive value and clinical utility of incorporating GLR. To explore the consistency of GLR's effect on in-hospital mortality, we conducted stratified analyses across various prespecified subgroups. All statistical analyses were performed using R software (version 4.3.2), and a two-sided P-value < 0.05 was considered statistically significant.

## Result

### Baseline characteristics

**Fig. 1:**
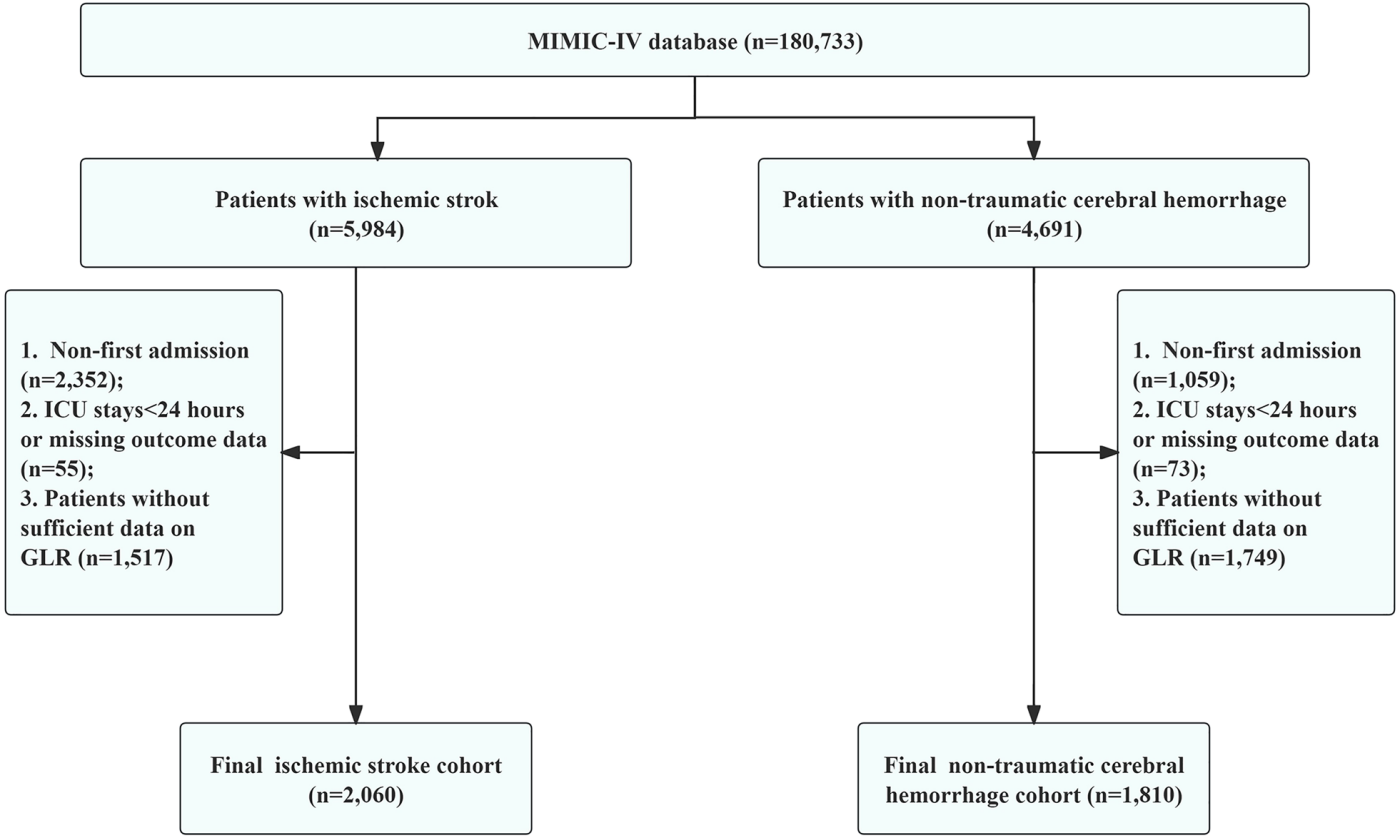
Flowchart of patient selection

As shown in Fig. 1, the final two cohorts of this study included 2060 patients with ischemic stroke and 1810 patients with non-traumatic cerebral hemorrhage from the MIMIC-IV database. Patients were divided into low and high GLR groups (GLR < 7.35 vs. ≥ 7.35 for ischemic stroke, n = 1037 vs. n = 1023; GLR < 7.035 vs. ≥ 7.035 for non-hemorrhagic stroke, n = 1214 vs. n = 596) through the optimal cutoff values. In the hemorrhagic stroke cohort, the median age was 69 years with 53.2% being male. In the ischemic stroke cohort, the median age was 71 years with 50.9% being male. Patients with higher GLR had longer hospital length of stay (9.08 vs. 7.96 days for hemorrhagic stroke; 8.62 vs. 7.03 days for ischemic stroke), and ICU length of stay (4.24 vs. 3.22 days for hemorrhagic stroke; 2.89 vs. 2.53 days for ischemic stroke), and higher in-hospital mortality (20.3% vs. 8.2% for hemorrhagic stroke; 15.6% vs. 8.1% for ischemic stroke). Detailed baseline characteristics of patients with non-traumatic cerebral hemorrhage and ischemic stroke, stratified by GLR, are provided in Table 1 and Table 2. The stratification by survival status is detailed in Supplementary Table 1 and Table 2.

**Table 1:** Characteristics of patients with non-traumatic cerebral hemorrhage

Characteristics	Overall (n = 1810)	Low GLR (< 7.035) n = 1214	High GLR (≥ 7.035) n = 596	*P*
Site (n, %)				
Cerebellum	66 (3.6)	33 (2.7)	33 (5.5)	0.01
Cortical	289 (16.0)	199 (16.4)	90 (15.1)	
Intraventricular	326 (18.0)	203 (16.7)	123 (20.6)	
Subarachnoid	205 (11.3)	137 (11.3)	68 (11.4)	
Subdural	290 (16.0)	218 (18.0)	72 (12.1)	
Other	634 (35.0)	424 (34.9)	210 (35.2)	
Male (n, %)	963 (53.2)	632 (52.1)	331 (55.5)	0.179
Age (y, IQR)	68 (57–79)	68 (56–79)	70 (59–80)	0.03
Race (n, %)				
Asian	77 (4.3)	50 (4.1)	27 (4.5)	< 0.01
Black	205 (11.3)	161 (13.3)	44 (7.4)	
White	1119 (61.8)	759 (62.5)	360 (60.4)	
Other	409 (22.6)	244 (20.1)	165 (27.7)	
Alcohol use (n, %)	180 (9.9)	109 (9)	71 (11.9)	0.061
Tobacco use (n, %)	267 (14.8)	201 (16.6)	66 (11.1)	0.03
Anemia (n, %)	533 (29.4)	334 (27.5)	199 (33.4)	0.012
CVD (n, %)	442 (24.4)	262 (21.6)	180 (30.2)	< 0.01
CKD (n, %)	342 (18.9)	1107 (17.1)	135 (22.7)	0.05
Diabetes (n, %)	497 (27.5)	273 (22.5)	224 (37.6)	< 0.01
Hyperlipemia (n, %)	947 (52.3)	634 (42.2)	313 (52.5)	0.946
Hypertension (n, %)	1209 (66.8)	809 (66.6)	400 (67.1)	0.882
Malignancy (n, %)	219 (12.1)	129 (10.6)	90 (15.1)	0.08
SBP (mmHg)	133 (118- 147)	132 (117–146)	134 (119–150)	0.016
DBP (mmHg)	72.0 (62.0–83.0)	73.0 (63.0–83.0)	71.0 (60.75–84.0)	0.122
Temperature (°C)	36.8 (36.5–37.1)	36.8 (36.6–37.1)	36.9 (36.6–37.2)	0.09
HR (beats/min)	81.0 (70.0–94.0)	80.0 (69.0–92.0)	84.0 (72.8–97.0)	< 0.01
RR (beats/min)	18.0 (15.0–21.0)	18.0 (15.0–21.0)	18.0 (16.0–22.0)	0.01
AST (IU/L)	26.0 (19.0–38.0)	25.0 (19.0–36.0)	27.0 (19.0–43.0)	0.03
ALT (IU/L)	21.0 (15.0–33.0)	21.0 (14.0–32.0)	22.0 (15.0–35.0)	0.029
Hemoglobin (g/dL)	13.1 (11.7–14.3)	13.2 (11.9–14.4)	12.7 (11.1–14.)	< 0.01
WBC (10^9^/L)	8.7 (6.8–12.0)	8.5 (6.8–11.5)	9.50 (6.8–13.1)	0.01
Platelet (10^9^/L)	216.0 (175.0–269.8)	221.0 (181.0–273.0)	203.0 (161.0–255.0)	< 0.01
INR	1.1 (1.0–1.2)	1.1 (1.0–1.2)	1.1 (1.0–1.3)	< 0.01
PTT (s)	28.5 (25.8–31.7)	28.7 (25.9–31.9)	28.2 (25.6–31.1)	0.017
Potassium (mmol/L)	4.1 (3.8–4.5)	4.1 (3.8–4.5)	4.2 (3.8–4.6)	0.08
Sodium (mmol/L)	139.0 (137.0–141.0)	140.0 (137.0–142.0)	139.0 (136.0–141.0)	< 0.01
Glucose (mmol/L)	6.56 (5.4–8.3)	5.97 (5.2–7.2)	8.28 (6.6–10.7)	< 0.01
Lymphocyte (10^9^/L)	1.3 (0.9–1.9)	1.6 (1.3–2.2)	0.7 (0.5–1.0)	< 0.01
GCS	15.0 (14.0–15.0)	15.0 (14.0–15.0)	15.0 (14.0–15.0)	0.46
SAPSII	32.0 (25.0–40.0)	31.0 (24.0–39.0)	34.5 (27.0–42.0)	< 0.01
OASIS	29.0 (24.0–35.0)	28.0 (23.0–34.0)	32.0 (26.0–37.0)	< 0.01
APSIII	33.0 (25.0–46.0)	31.0 (24.0–43.0)	38.0 (28.0–51.0)	< 0.01
Hospital length of stay (day)	8.4 (4.6–15.6)	8.0 (4.4–14.0)	9.1 (5.1–17.4)	< 0.01
ICU length of stay (day)	3.5 (1.8- 7.6)	3.2 (1.7- 7.0)	4.2 (2.0- 9.0)	< 0.01

Abbreviation: GLR, glucose-to-lymphocyte ratio; CVD, cardiovascular disease; CKD, chronic kidney disease; SBP, systolic blood pressure; DBP, diastolic blood pressure; HR, heart rate; RR, respiratory rate; AST, aspartate aminotransferase; ALP, alkaline phosphatase; ALT, alanine aminotransferase; WBC, white blood cell count; INR, international normalized ratio; PTT, partial thromboplastin time; GCS, Glasgow coma scale; SAPSII, simplified acute physiology score II; OASIS, oxford acute severity of illness score; APSIII, acute physiology score III

**Table 2:** Characteristics of patients with ischemic stroke

Characteristics	Overall (n = 2060)	Low GLR (< 7.35) n = 1037	High GLR (≥ 7.35) n = 1023	*P*
Site (n, %)				
Anterior cerebral artery	34 (1.7)	16 (1.5)	18 (1.8)	0.525
Carotid artery	140 (6.8)	69 (6.7)	71 (6.9)	
Cerebellar artery	124 (6.0)	63 (6.1)	61 (6.0)	
Middle cerebral artery	477 (23.2)	255 (24.6)	222 (21.7)	
Posterior cerebral artery	76 (3.7)	32 (3.1)	44 (4.3)	
Vertebrobasilar artery	46 (2.2)	26 (2.5)	20 (2.0)	
Other	1163 (56.5)	576 (55.5)	587 (57.4)	
Male (n, %)	1049 (50.9)	488 (47.1)	561 (54.8)	< 0.001
Age (y, IQR)	71 (60- 81)	69 (59- 81)	72 (61- 82)	0.032
Race (n, %)				
Asian	73 (3.5)	44 (4.2)	29 (2.8)	0.026
Black	308 (15.0)	169 (16.3)	139 (13.6)	
Other	442 (21.5)	232 (22.4)	210 (20.5)	
White	1237 (60.0)	592 (57.1)	645 (63.0)	
Alcohol use (n, %)	195 (9.5)	106 (10.2)	89 (8.7)	0.269
Tobacco use (n, %)	385 (18.7)	212 (20.4)	173 (16.9)	0.046
Anemia (n, %)	812 (39.4)	400 (38.6)	412 (40.3)	0.456
CVD (n, %)	618 (30.0)	286 (27.6)	332 (32.5)	0.018
CKD (n, %)	871 (42.3)	410 (39.5)	461 (45.1)	0.013
Diabetes (n, %)	842 (40.9)	366 (35.3)	476 (46.5)	< 0.001
Hyperlipemia (n, %)	1412 (68.5)	732 (70.6)	680 (66.5)	0.049
Hypertension (n, %)	1378 (66.9)	720 (69.4)	658 (64.3)	0.016
Malignancy (n, %)	225 (90.9)	98 (9.5)	127 (12.4)	0.037
SBP (mmHg)	133 (116–152)	133 (117–151)	133 (115–153)	0.806
DBP (mmHg)	72 (61–85)	73 (61–85)	72 (61–85)	0.467
Temperature (°C)	36.8 (36.6–37.1)	36.8 (36.5–37.1)	36.8 (36.5–37.0)	0.928
HR (beats/min)	82 (72–96)	82 (71–95)	83 (73–97)	0.019
RR (beats/min)	18 (16–22)	18 (15–22)	19 (16–23)	0.061
AST (IU/L)	25.0 (19.0–37.3)	24.0 (19.0–35.0)	25.0 (19.0–42.0)	0.005
ALT (IU/L)	21 (14–32)	20 (14–30)	22 (15–36)	< 0.001
Hemoglobin (g/dL)	12.9 (11.5–14.2)	13.0 (11.7–14.2)	12.8 (11.1–14.0)	0.001
WBC (10^9^/L)	8.6 (6.6–11.6)	8.5 (6.6–11.1)	8.8 (6.7–12.0)	0.054
Platelet (10^9^/L)	228 (180- 284)	235 (186- 286)	219 (173- 282)	0.001
INR	1.1 (1.0–1.2)	1.1 (1.0–1.2)	1.1 (1.0–1.3)	< 0.001
PTT (s)	28.7 (25.8–32.1)	28.8 (26.1–32.1)	28.6 (25.7–32.1)	0.276
Potassium (mmol/L)	4.2 (3.9–4.6)	4.2 (3.9–4.5)	4.2 (3.9–4.7)	0.008
Sodium (mmol/L)	139 (137–142)	140 (137–142)	139 (136–141)	< 0.001
Glucose (mmol/L)	6.5 (5.4–8.6)	6.1 (5.2–7.4)	7.2 (5.7–10.4)	< 0.001
Lymphocyte (10^9^/L)	1.4 (0.9–2.0)	1.7 (1.3–2.3)	1.1 (0.7–1.6)	< 0.001
GCS	15.0 (14.0–15.0)	15.0 (14.0–15.0)	15.0 (14.0–15.0)	0.265
SAPSII	33.0 (26.0–42.0)	32.0 (25.0–40.0)	35.0 (27.0–43.5)	< 0.001
OASIS	30.0 (24.0–36.0)	30.0 (24.0–36.0)	31.0 (25.0–37.0)	0.002
APSIII	37.0 (28.0–50.0)	35.0 (26.0–46.0)	40.0 (29.0–53.0)	< 0.001
Hospital length of stay (day)	7.8 (4.4–14.7)	7.0 (4.1–13.1)	8.6 (4.8–16.7)	< 0.001
ICU length of stay (day)	2.7 (1.5–5.7)	2.5 (1.4–5.0)	2.9 (1.6–6.7)	< 0.001

Abbreviation: GLR, glucose-to-lymphocyte ratio; CVD, cardiovascular disease; CKD, chronic kidney disease; SBP, systolic blood pressure; DBP, diastolic blood pressure; HR, heart rate; RR, respiratory rate; AST, aspartate aminotransferase; ALP, alkaline phosphatase; ALT, alanine aminotransferase; WBC, white blood cell count; INR, international normalized ratio; PTT, partial thromboplastin time; GCS, Glasgow coma scale; SAPSII, simplified acute physiology score II; OASIS, oxford acute severity of illness score; APSIII, acute physiology score III

### Association of GLR index with the severity of consciousness disturbance and in-hospital mortality

To elucidate the potential relationship between the GLR and outcomes in patients with non-traumatic intracerebral hemorrhage and ischemic stroke, cox proportional hazards regression was used to analyze the association between GLR and in-hospital mortality. For the secondary outcome, severe consciousness disturbance, binary logistic regression was employed to assess its association with GLR (Table 3).

**Table 3:** Association between GLR and clinical outcomes in patients with cerebrovascular disease

Categories	Unadjusted		Model 1		Model 2	
	HR/OR^1^ (95% CI)	P-value	HR/OR (95% CI)	P-value	HR/OR (95% CI)	P-value
Patients with non-traumatic cerebral hemorrhage						
In-hospital mortality						
Continuous variable per unit, GLR^2^	1.020 (1.011, 1.030)	< 0.001	1.019 (1.009, 1.029)	< 0.001	1.016 (1.005, 1.027)	0.004
GLR (< 7.085)	1 (Ref)					
GLR (≥ 7.085)	2.162 (1.656, 2.822)	< 0.001	1.995 (1.526, 2.610)	< 0.001	1.918 (1.451, 2.536)	< 0.001
Severe consciousness disturbance (GCS ≤ 8)						
Continuous variable per unit, GLR^2^	1.021 (1.008, 1.033)	< 0.001	1.017 (1.005, 1.030)	0.007	1.014 (1.000, 1.027)	0.043
GLR (< 7.085)	1 (Ref)					
GLR (≥ 7.085)	1.884 (1.492, 2.377)	< 0.001	1.775 (1.399, 2.250)	< 0.001	1.653 (1.284, 2.126)	< 0.001
Patients with ischemic stroke						
In-hospital mortality						
Continuous variable per unit, GLR^2^	1.013 (1.004, 1.023)	0.005	1.014 (1.004, 1.024)	0.005	1.013 (1.003, 1.022)	0.009
GLR (< 7.350)	1 (Ref)					
GLR (≥ 7.350)	1.414 (1.068, 1.871)	0.015	1.513 (1.148, 1.994)	0.003	1.546 (1.175, 2.035)	0.002
Severe consciousness disturbance (GCS ≤ 8)						
Continuous variable per unit, GLR^2^	1.019 (1.008, 1.030)	< 0.001	1.020 (1.009, 1.031)	< 0.001	1.015 (1.003, 1.027)	0.013
GLR (< 7.350)	1 (Ref)					
GLR (≥ 7.350)	1.681 (1.339, 2.107)	< 0.001	1.676 (1.328, 2111)	< 0.001	1.517 (1.182, 1.943)	0.001

Abbreviations: GLR, glucose-to-lymphocyte ratio; HR, hazard ratio; OR, odds ratio; CI, confidence interval; GCS, Glasgow Coma Scale; Ref, reference

Model 1: Adjusted for age, gender, and race

Model 2: Adjusted for age, sex, race, temperature, SBP, ALT, AST, HbA1c, platelet, PTT, INR, WBC, sodium, potassium, CKD, CVD, hyperlipemia, hypertension, malignancy, diabetes, anemia, alcohol use, tobacco use

HR/OR^1^: HR was reported for in-hospital mortality and OR was reported for severe consciousness disturbance;

GLR^2^: Per 1-unit increase in GLR for continuous analysis

The analysis of patients with non-traumatic intracerebral hemorrhage demonstrated that elevated GLR was associated with increased risks of both in-hospital mortality and severe consciousness disturbance across all three models. For continuous GLR, each unit increase significantly elevated the risk of in-hospital mortality in the unadjusted model (HR: 1.020, 95% CI: 1.011–1.030, *P* < 0.001), Model 1 (HR: 1.019, 95% CI: 1.009–1.029, *P* < 0.001), and Model 2 (HR: 1.016, 95% CI: 1.005–1.027, *P* = 0.004). Similarly, GLR as a categorical variable (GLR ≥ 7.085 vs. GLR < 7.085) showed a consistent trend, with high GLR patients exhibiting greater risks of in-hospital mortality in the unadjusted model (HR: 2.162, 95% CI: 1.656–2.822, *P* < 0.001), Model 1 (HR: 1.995, 95% CI: 1.526–2.610, *P* < 0.001), and Model 2 (HR: 1.918, 95% CI: 1.451–2.536, *P* < 0.001). For severe consciousness impairment, continuous GLR was significantly associated with higher risks in the unadjusted model (OR: 1.021, 95% CI: 1.008–1.033, *P* < 0.001), Model 1 (OR: 1.017, 95% CI: 1.005–1.030, P = 0.007), and Model 2 (OR: 1.014, 95% CI: 1.000–1.027, *P* = 0.043). Similarly, a categorical GLR (GLR ≥ 7.085) was linked to significantly increased risks of severe consciousness impairment in the unadjusted model (OR: 1.884, 95% CI: 1.492–2.377, *P* < 0.001), Model 1 (OR: 1.775, 95% CI: 1.399–2.250, *P* < 0.001), and Model 2 (OR: 1.653, 95% CI: 1.284–2.126, *P* < 0.001).

For ischemic stroke patients, elevated GLR levels were independently associated with higher risks of in-hospital mortality and severe consciousness disturbance. Specifically, each unit increase in GLR significantly raised the risk of in-hospital mortality (HR: 1.013, 95% CI: 1.003–1.022, *P* = 0.009) and severe consciousness disturbance (OR: 1.015, 95% CI: 1.003–1.027, *P* = 0.013) in the fully adjusted model. When analyzed as a categorical variable (GLR ≥ 7.35 vs. GLR < 7.35), high GLR levels were consistently linked to greater in-hospital mortality risk across all models: unadjusted (HR: 1.414, 95% CI: 1.068–1.871, *P* = 0.015), partially adjusted (HR: 1.513, 95% CI: 1.148–1.994, *P* = 0.003), and fully adjusted (HR: 1.546, 95% CI: 1.175–2.035, *P* = 0.002). Similarly, patients with GLR ≥ 7.35 exhibited significantly elevated risks of severe consciousness disturbance (OR: 1.78, 95% CI: 1.23–2.56, *P* = 0.004) in the fully adjusted model.

**Fig. 2:**
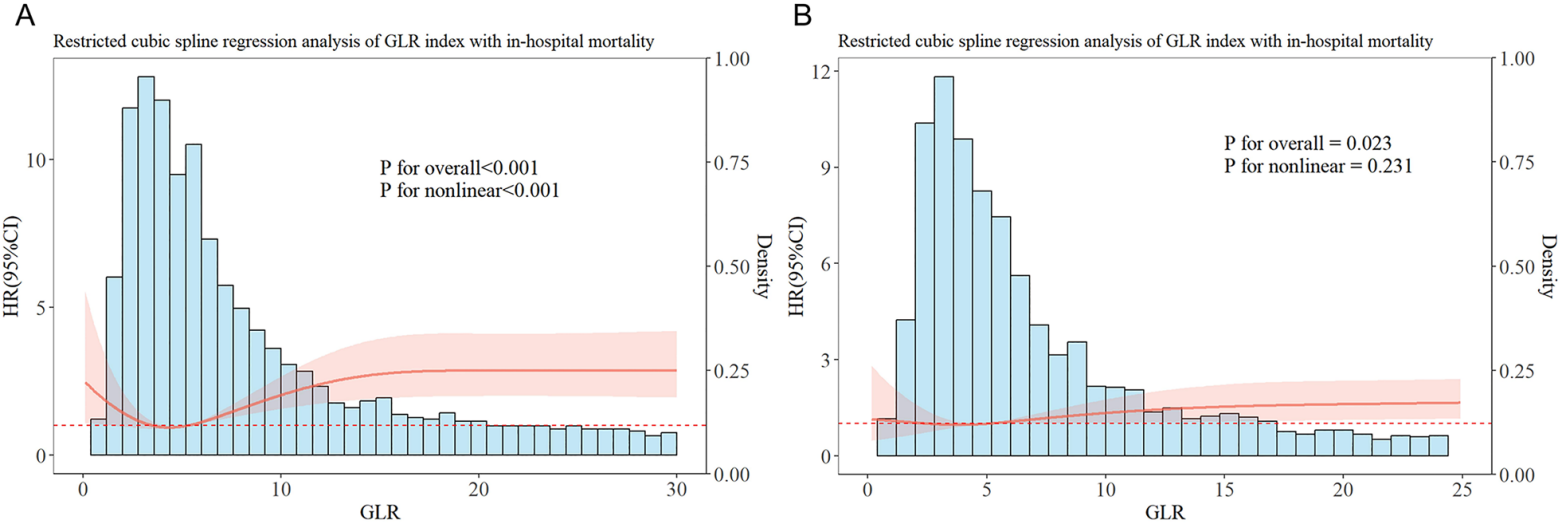
RCS analysis of the association between GLR and in-hospital mortality. **A** Association between GLR and in-hospital mortality in patients with non-traumatic cerebral hemorrhage; **B** Association between GLR and in-hospital mortality in patients with ischemic stroke

Additionally, restricted cubic spline regression was used to explore the non-linear association between GLR and in-hospital mortality in the two cohorts (Fig. 2). In patients with non-traumatic cerebral hemorrhage, a significant non-linear association was observed between GLR and in-hospital mortality (*P* for non-linearity < 0.001). When GLR exceeded 5, the risk of in-hospital mortality increased sharply, as reflected by the upward slope of the RCS curve, demonstrating a J-shaped relationship (Fig. 2A). On the other hand, ischemic stroke patients showed a significant but relatively linear association (P for overall = 0.023, *P* for non-linear = 0.231) (Fig. 2B).

**Fig. 3:**
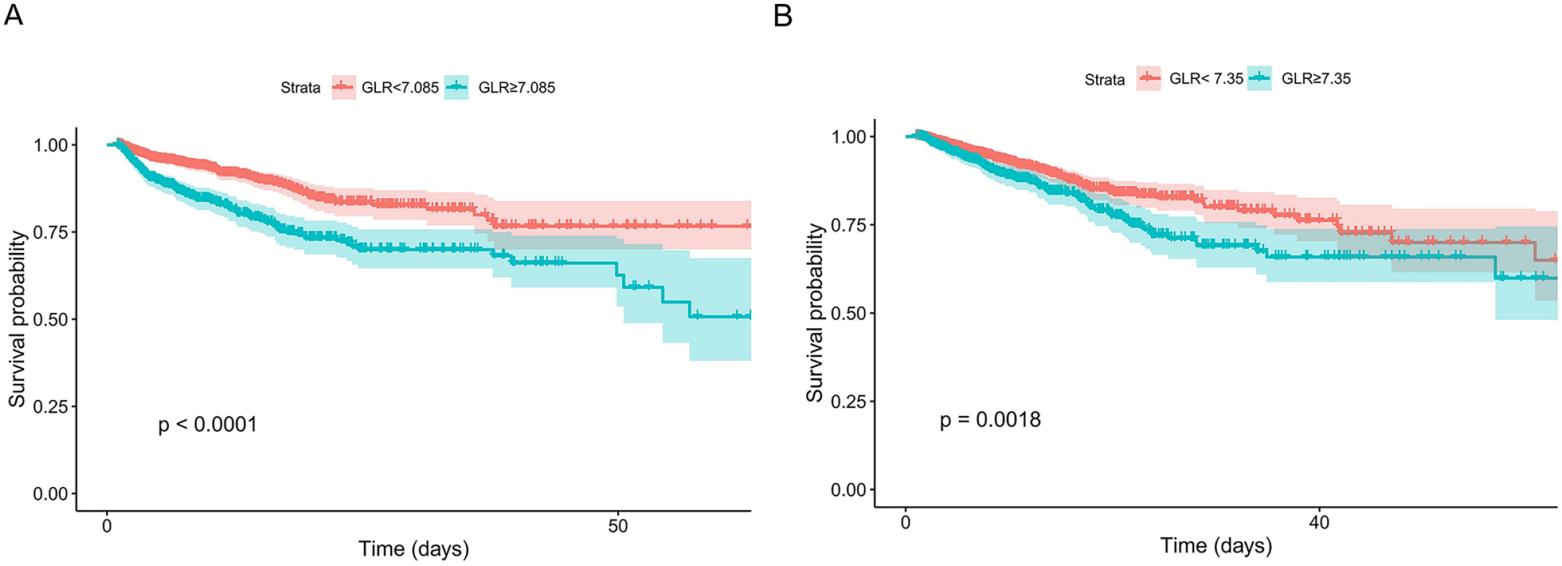
Kaplan–Meier survival curves. **A** For patients with non-traumatic cerebral hemorrhage; **B** for patients with ischemic stroke

To further validate these findings, we performed survival analysis using Kaplan–Meier curves (Fig. 3). Patients were stratified into two groups based on GLR. In the non-traumatic cerebral hemorrhage cohort, patients with GLR ≥ 7.085 exhibited significantly worse survival compared to those with lower GLR levels (*P* < 0.001). Similarly, in the ischemic stroke group, a significant difference in survival was also observed between the two GLR groups (*P* = 0.0018).

### The predictive performance and clinical utility analysis of GLR

**Fig. 4:**
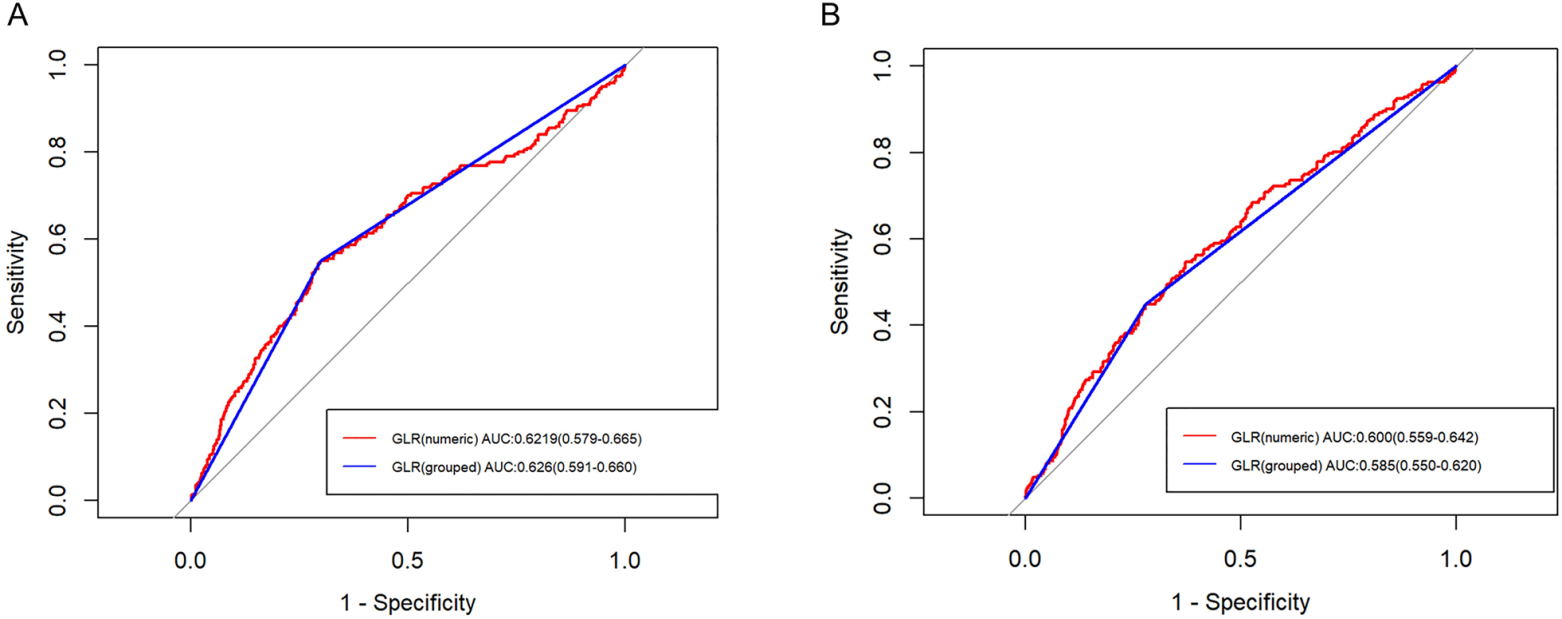
ROC curves of GLR for predicting in-hospital mortality. **A** For patients with non-traumatic cerebral hemorrhage; **B** for patients with ischemic stroke

The predictive value of GLR on in-hospital mortality was evaluated using ROC curve analysis (Fig. 4). In non-traumatic cerebral hemorrhage patients, both numeric and grouped GLR demonstrated the ability to provide meaningful predictive value, as presented with area under the curve (AUC) values [grouped: 0.626 (95% CI: 0.591–0.660); numeric: 0.622 (95% CI: 0.579–0.665)]. Similarly, in ischemic stroke patients, the AUCs were 0.600 (95% CI: 0.559–0.642) for numeric GLR and 0.585 (95% CI: 0.550–0.620) for grouped GLR.

The addition of GLR to existing severity scores was assessed using IDI, a metric that evaluates the degree of improvement in a model's predictive ability (Table 4). An IDI value greater than 0 indicates a positive enhancement, while a value less than 0 suggests a decrease in predictive performance. In non-traumatic cerebral hemorrhage patients, grouped GLR significantly improved the predictive performance of all three scoring systems: APSIII (*P* = 0.004), OASIS (*P* < 0.001), and SAPSII (*P* < 0.001). For ischemic stroke patients, similar significant improvements were observed when adding grouped GLR to APSIII (*P* = 0.026), OASIS (*P* = 0.021), and SAPSII (*P* = 0.022). However, the addition of numeric GLR showed no significant improvement in predictive performance for either stroke subtype (all *P* > 0.05).

**Table 4:** The incremental effect of the GLR index

Score	AUC [95% CI]	IDI [95% CI] (+ GLR (grouped))	*P*-value	IDI [95% CI] (+ GLR (numeric))	*P*-value
In-hospital mortality (patients with non-traumatic cerebral hemorrhage)					
APSIII	0.711 (0.672, 0.751)	0.014 (0.004, 0.023)	0.004	0.003 (− 0.002, 0.009)	0.188
OASIS	0.765 (0.737, 0.797)	0.021 (0.011, 0.031)	< 0.001	0.006 (− 0.001, 0.012)	0.053
SAPSII	0.746 (0.713, 0.778)	0.017 (0.008, 0.026)	< 0.001	0.005 (0.001, 0.010)	0.106
In-hospital mortality (patients with ischemic stroke)					
APSIII	0.714 (0.677, 0.750)	0.006 (0.001, 0.011)	0.026	0.003 (− 0.002–0.008)	0.257
OASIS	0.737 (0.703, 0.771)	0.006 (0.001, 0.010)	0.021	0.005 (− 0.001, 0.012)	0.119
SAPSII	0.748 (0.715, 0.780)	0.006 (0.001, 0.012)	0.022	0.005 (− 0.002, 0.011)	0.163

Abbreviations: GLR, glucose-to-lymphocyte ratio; AUC, area under the curve; CI, confidence interval; IDI, integrated discrimination improvement; APSIII, Acute Physiology and Chronic Health Evaluation III; OASIS, Oxford Acute Severity of Illness Score; SAPSII, Simplified Acute Physiology Score II

AUC values were calculated for original scoring systems;

IDI values represent the improvement in discrimination when adding GLR (grouped or numeric) to each scoring system

Furthermore, decision curve analysis assessed the clinical utility of adding GLR to conventional scoring systems (Fig. S1). In both cohorts, the combination of GLR with existing severity scores (APSIII, OASIS, and SAPSII) showed consistent net benefit across various threshold probabilities compared to the original scores alone.

### Subgroup analysis

**Fig. 5:**
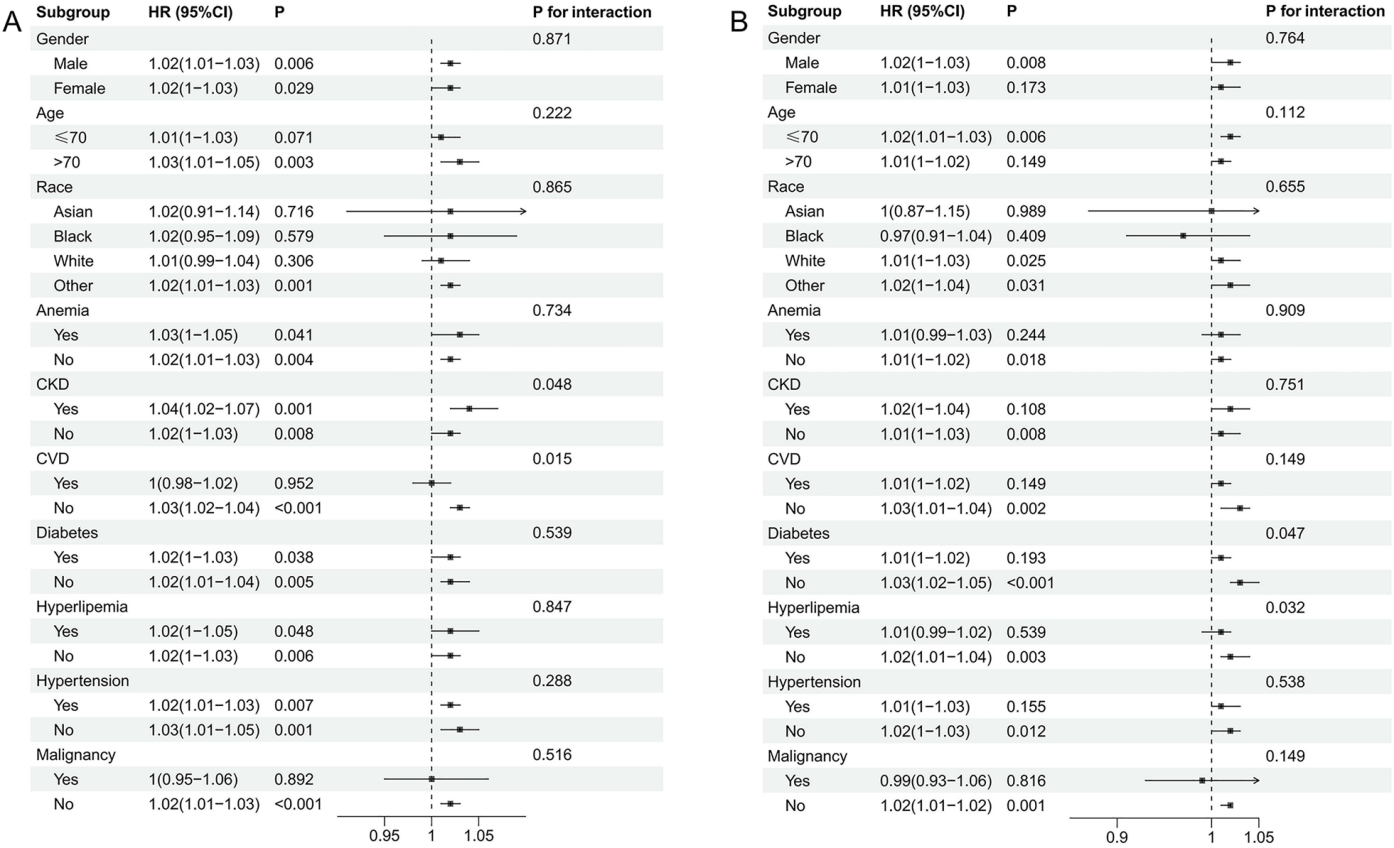
Subgroup analysis for the effect of GLR on in-hospital mortality in patients with **A** non-traumatic cerebral hemorrhage; **B** ischemic stroke

In non-traumatic cerebral hemorrhage patients, consistent associations between GLR and mortality were observed across subgroups of gender, age, race, anemia, diabetes, hyperlipemia, hypertension, and malignancy, with no significant interactions detected for these variables. The associations were particularly pronounced in patients without malignancy (*P* < 0.001) and without hypertension (*P* = 0.001). Notably, significant interactions were found for CKD and CVD (*P* for interaction = 0.048 and 0.015, respectively). In patients with CKD, GLR showed a stronger positive correlation with mortality (HR: 1.04, 95%CI: 1.02–1.07, *P* = 0.001). For CVD, a significant association was observed in patients without CVD (HR: 1.03, 95%CI: 1.02–1.04, *P* < 0.001), while no significant association was found in patients with CVD (Fig. 5A).

For ischemic stroke patients, the associations between GLR and mortality remained consistent across subgroups of gender, age, race, anemia, diabetes, hyperlipemia, hypertension, and malignancy, without significant interactions. Significant interactions were found for diabetes and hyperlipemia (*P* for interaction = 0.047 and 0.032, respectively). The prognostic value of GLR demonstrated varying effects in these subgroups, with stronger associations observed in patients without diabetes (HR: 1.03, 95%CI: 1.02–1.05, *P* < 0.001) and without hyperlipemia (HR: 1.02, 95%CI: 1.01–1.04, *P* = 0.003) (Fig. 5B).

## Discussion

In this comprehensive analysis of critically ill patients with cerebrovascular diseases, we demonstrated that elevated GLR was associated with increased in-hospital mortality risk and severe consciousness impairment. Notably, the relationship between GLR and mortality exhibited distinct patterns in different stroke subtypes, with a non-linear J-shaped association in hemorrhagic stroke. Furthermore, the integration of GLR enhanced the predictive performance of conventional clinical scoring systems, suggesting its potential value in risk stratification. These findings highlight GLR as a readily available and promising prognostic marker in cerebrovascular diseases.

Previous research has established that inflammation and hyperglycemia contribute to the pathophysiological processes associated with cerebrovascular disease [20, 21]. Blood glucose levels play a crucial role in cerebrovascular diseases through multiple pathways [22]. Hyperglycemia not only disrupts energy metabolism and increases oxidative stress but also impairs vascular endothelial function, leading to microcirculatory disturbances and tissue hypoxia [23, 24]. Furthermore, elevated glucose levels enhance inflammatory cytokine secretion, potentially exacerbating tissue damage [25]. Similarly, lymphocytes serve as key indicators of immune status and inflammatory response, with reduced lymphocyte counts often reflecting stress-induced immunosuppression [26]. This immunosuppressive state may increase susceptibility to complications and impair tissue repair processes, further contributing to adverse outcomes [27]. Indeed, clinical evidence has demonstrated that both hyperglycemia and lymphopenia independently correlate with poor outcomes in cerebrovascular diseases [20, 28, 29]. In this context, The GLR represents a biomarker that integrates inflammatory indicators, including lymphocyte count and blood glucose levels for prognostic value in cerebrovascular diseases. GLR has been established as an independent prognostic factor in various malignancy [30–33]. Another study found a significant correlation between higher GLR (grouped according to the upper quartile) and increased mortality risk of non-traumatic intracerebral hemorrhage [34]. Most studies focused on the neutrophil-to-lymphocyte ratio (NLR) and its effect on the prognosis of intracerebral hemorrhage and cerebral infarction [35, 36]. The exact mechanism underlying the association between elevated GLR levels and poor prognosis remains unclear. We found that GLR was an independent risk factor for disturbance of consciousness and hospital mortality either as a continuous or categorical variable. These findings align with prior studies highlighting the prognostic roles of hyperglycemia and lymphopenia in various critical conditions, underscoring the relevance of GLR as an integrated marker of metabolic and immune status [37, 38]. Notably, while GLR reflects metabolic-immune dysfunction, anatomical factors such as intraventricular hemorrhage extension remain critical prognostic determinants in ICH that warrant consideration in comprehensive risk assessment [39]. Our study demonstrated that incorporating GLR into established clinical models, such as APSIII, OASIS, and SAPSII, enhanced their predictive accuracy. This finding suggests that GLR may serve as a valuable tool for risk stratification and clinical decision-making in patients with cerebrovascular diseases.

The GLR index reflects not merely hyperglycemia or heightened inflammation but rather an imbalance between glucose regulation and immune response [34]. This imbalance can drive organ failure, metabolic disturbances, compromised immune function, and insufficient oxygen utilization, ultimately culminating in mortality. However, our study could not distinguish whether elevated GLR primarily predicts death from direct neurological complications or secondary systemic complications, which warrants investigation in future studies. In our study, the relationship between GLR and outcomes varied by stroke type, with a J-shaped non-linear association between GLR and mortality in hemorrhagic stroke patients. This non-linearity may reflect a complex interaction where hyperglycemia exacerbates brain injury through oxidative stress and vascular damage, while lymphopenia reflects impaired immune recovery, further complicating outcomes. In contrast, ischemic stroke exhibited a more linear association between GLR and mortality, where hyperglycemia directly contributes to infarct growth and inflammation-induced neuronal injury. These disparate patterns underscore the need for further mechanistic studies to elucidate the precise pathways through which GLR influences outcomes in different stroke subtypes.

Subgroup analyses revealed significant interactions between GLR and certain comorbidities. Although pre-existing conditions such as diabetes, malignancy, and anemia could theoretically influence GLR components, our subgroup analyses demonstrated that GLR maintained its prognostic value across most patient populations, with consistent associations observed in patients without these conditions and varying patterns in those with these comorbidities. In non-traumatic intracerebral hemorrhage patients, the association between GLR and mortality was particularly pronounced in those with CKD, likely due to the heightened inflammation and metabolic derangements associated with impaired kidney function [40]. Interestingly, while elevated GLR was significantly linked to higher mortality risk among patients without CVD, this association was not observed in those with CVD. This difference may be attributable to the fact that CVD patients often exhibit chronic inflammation and dysregulated glucose metabolism at baseline, which could potentially mask the predictive value of GLR [41, 42]. Similarly, in ischemic stroke patients without diabetes or hyperlipidemia, elevated GLR is strongly associated with higher mortality risk. Given that diabetes and hyperlipidemia are chronic conditions characterized by persistent inflammation and metabolic imbalances [43, 44], these factors might attenuate the predictive sensitivity of GLR regarding mortality risk. The presence of long-standing metabolic derangements in patients with diabetes or hyperlipidemia may create a ceiling effect, limiting the ability of GLR to further discriminate risk in these subpopulations.

There are some limitations to this study. Firstly, this single-center retrospective study may be prone to selection bias and limited generalizability beyond the American population. Secondly, due to database constraints, we could not adjust for important radiological factors including intraventricular hemorrhage extension and some potential clinical confounders such as dietary habits, physical activity levels, and Body Mass Index (BMI). Furthermore, the GLR values employed in this study were derived from static measurements conducted on the first day of admission, rather than dynamic assessments spanning the entirety of the illness. Future prospective studies across diverse populations are warranted to validate GLR’s role and to explore its integration with neuroimaging biomarkers, investigate underlying mechanisms, and evaluate whether targeted interventions addressing metabolic-immune dysfunction can improve outcomes.

## Conclusion

In conclusion, GLR is a promising, easily measurable biomarker for assessing prognosis in cerebrovascular disease patients, with significant associations in non-traumatic intracerebral hemorrhage and ischemic stroke. GLR could potentially improve early risk stratification and clinical decision-making, particularly in high-risk patients.

## Supplementary Information

Additional file 1.

## Data Availability

The datasets generated and analyzed in this study are available upon reasonable request to the corresponding author and require approval from the MIMIC database administrators.
