# Supplementary material for: Prognostic value of glucose-to-lymphocyte ratio for all-cause mortality and consciousness impairment in critically ill cerebrovascular disease patients
Source: Eur J Med Res. 2025 Nov 28;30:1189. doi: 10.1186/s40001-025-03378-6 (PMC12661742; doi:10.1186/s40001-025-03378-6)

Supplementary Table 1.

Baseline characteristics of patients with non-traumatic cerebral hemorrhage stratified by survival status.

| Characteristics | Overall  (n=1810) | Survivor  (n=1590) | Non-survivor  (n=220) | P |
| --- | --- | --- | --- | --- |
| Site (%) |  |  |  |  |
| Cerebellum | 66 (3.6) | 56 (3.5) | 10 (4.5) | 0.016 |
| Cortical | 289 (16.0) | 253 (15.9) | 36 (16.4) |  |
| Intraventricular | 326 (18.0) | 285 (17.9) | 41 (18.6) |  |
| Other | 634 (35.0) | 540 (34.0) | 94 (42.7) |  |
| Subarachnoid | 205 (11.3) | 186 (11.7) | 19 (8.6) |  |
| Subdural | 290 (16.0) | 270 (17.0) | 20 (9.1) |  |
| Male (n, %) | 963 (53.2) | 849 (53.4) | 114 (51.8) | 0.713 |
| Age (y, IQR) | 68.42 (56.69- 79.41) | 67.92 (56.25- 78.58) | 75.18 (62.16- 83.26) | <0.001 |
| Race (n, %) |  |  |  |  |
| Asian | 77 (4.3) | 66 (4.2) | 11 (5.0) | <0.001 |
| Black | 205 (11.3) | 190 (11.9) | 15 (6.8) |  |
| White | 1119 (61.8) | 1012 (63.6) | 107 (48.6) |  |
| Other | 409 (22.6) | 322 (20.3) | 87 (39.5) |  |
| Alcohol use (n, %) | 180 (9.9) | 153 (9.6) | 27 (12.3) | 0.267 |
| Tobacco use (n, %) | 267 (14.8) | 248 (15.6) | 19 (8.6) | 0.009 |
| Anemia (n, %) | 533 (29.4) | 485 (30.5) | 48 (21.8) | 0.01 |
| CVD (n, %) | 442 (24.4) | 377 (23.7) | 65 (29.5) | 0.071 |
| CKD (n, %) | 342 (18.9) | 297 (18.7) | 45 (20.5) | 0.59 |
| Diabetes (n, %) | 497 (27.5) | 435 (27.4) | 62 (28.2) | 0.86 |
| Hyperlipemia (n, %) | 947 (52.3) | 862 (54.2) | 85 (38.6) | <0.001 |
| Hypertension (n, %) | 1209 (66.8) | 1070 (67.3) | 139 (63.2) | 0.255 |
| Respiratory (n, %) | 546 (30.2) | 400 (25.2) | 146 (66.4) | <0.001 |
| Malignancy (n, %) | 219 (12.1) | 200 (12.6) | 19 (8.6) | 0.116 |
| SBP (mmHg) | 133 (118- 147) | 133 (118- 147) | 130 (114- 148) | 0.304 |
| DBP (mmHg) | 72.0 (62.0- 83.0) | 72.0 (62.25- 83.0) | 70.50 (59.0- 82.25) | 0.044 |
| AST (IU/L) | 26.0 (19.0- 38.0) | 25.0 (19.0- 36.0) | 32.0 (23.0- 48.4) | <0.001 |
| ALT (IU/L) | 21.0 (15.0- 33.0) | 21.0 (15.0- 32.0) | 22.0 (15.0- 35.3) | 0.22 |
| Glucose (mmol/L) | 6.6 (5.4- 8.3) | 6.44 (5.4- 8.1) | 7.47 (5.8- 9.9) | <0.001 |
| Hemoglobin (mmol/L) | 13.1 (11.7- 14.3) | 13.1 (11.7- 14.3) | 12.7 (11.1- 13.9) | 0.001 |
| WBC (10^9^/L) | 8.7 (6.8- 12.0) | 8.6 (6.7- 11.7) | 10.0 (7.4- 13.9) | <0.001 |
| Lymphocyte (10^9^/L) | 1.3 (0.9- 1.9) | 1.4 (0.9- 1.9) | 1.0 (0.6- 1.7) | <0.001 |
| Platelet (10^9^/L) | 216.0 (175.0- 269.8) | 218.0 (177.0- 272.0) | 193.5 (152.8- 246.0) | <0.001 |
| Potassium (mmol/L) | 4.1 (3.8- 4.5) | 4.1 (3.8- 4.5) | 4.1 (3.8- 4.6) | 0.984 |
| Sodium (mmol/L) | 139.0 (137.0- 141.0) | 139.0 (137.0- 141.0) | 139.0 (136.0- 142.0) | 0.548 |
| Temperature (°C) | 36.8 (36.6- 37.1) | 36.8 3 (36.6- 37.1) | 36.7 (36.5- 37.2) | 0.933 |
| HR (beats/min) | 81 (70- 94) | 81 (70- 93) | 83 (72- 96) | 0.026 |
| PTT (s) | 28.5 (25.8- 31.7) | 28.5 (25.8- 31.6) | 28.5 (26.1- 32.3) | 0.233 |
| RR (beats/min)) | 18 (15- 21) | 18 (15- 21) | 19 (16- 23) | <0.001 |
| INR | 1.1 (1.0- 1.2) | 1.1 (1.0- 1.2) | 1.1 (1.0- 1.4) | <0.001 |
| GCS | 15.0 (14.0- 15.0) | 15.0 (14.0- 15.0) | 15.0 (14.0- 15.0) | 0.816 |
| SAPSII (median (IQR)) | 32.0 (25.0- 40.0) | 31.0 (24.0- 38.0) | 40.0 (33.75- 51.0) | <0.001 |
| OASIS | 29.0 (24.0- 35.0) | 29.0 (23.0- 34.0) | 37.0 (32.0- 42.3) | <0.001 |
| APSIII | 33.0 (25.0- 46.0) | 32.0 (24.0- 43.0) | 47.0 (33.8- 61.3) | <0.001 |
| Hospital length of stay (day) | 8.4 (4.6- 15.6) | 8.7 (4.9- 15.8) | 5.7 (2.8- 13.1) | <0.001 |
| ICU length of stay (day) | 3.5 (1.8- 7.6) | 3.5 (1.8- 7.5) | 3.7 (2.0- 8.2) | 0.188 |

Abbreviation: GLR, glucose-to-lymphocyte ratio; CVD, cardiovascular disease; CKD, chronic kidney disease; SBP, systolic blood pressure; DBP, diastolic blood pressure; HR, heart rate; RR, respiratory rate; AST, aspartate aminotransferase; ALP, alkaline phosphatase; ALT, alanine aminotransferase; WBC, white blood cell count; INR, international normalized ratio; PTT, partial thromboplastin time; GCS, Glasgow coma scale; SAPSII, simplified acute physiology score II; OASIS, oxford acute severity of illness score; APSIII, acute physiology score III.

Supplementary Table 2.

Baseline characteristics of patients with ischemic stroke stratified by survival status.

| Characteristics | Overall  (n=2060) | Survivor  (n=1848) | Non-survivor  (n=212) | P |
| --- | --- | --- | --- | --- |
| Site (%) |  |  |  |  |
| Anterior cerebral artery | 34 (1.7) | 28 (1.5) | 6 (2.8) | 0.006 |
| Carotid artery | 140 (6.8) | 123 (6.7) | 17 (8.0) |  |
| Cerebellar artery | 124 (6.0) | 117 (6.3) | 7 (3.3) |  |
| Middle cerebral artery | 477 (23.2) | 421 (22.8) | 56 (26.4) |  |
| Posterior cerebral artery | 76 (3.7) | 60 (3.2) | 16 (7.5) |  |
| Vertebrobasilar artery | 46 (2.2) | 42 (2.3) | 4 (1.9) |  |
| Other | 1163 (56.5) | 1057 (57.2) | 106 (50.0) |  |
| Male (n, %) | 1049 (50.9) | 944 (51.1) | 105 (49.5) | 0.722 |
| Age (y, IQR) | 71 (60- 81) | 70 (59- 80) | 75 (65- 85) | <0.001 |
| Race (%) |  |  |  |  |
| Asian | 73 (3.5) | 65 (3.5) | 8 (3.8) | <0.001 |
| Black | 308 (15.0) | 280 (15.2) | 28 (13.2) |  |
| White | 1237 (60.0) | 1134 (61.4) | 103 (48.6) |  |
| Other | 442 (21.5) | 369 (20.0) | 73 (34.4) |  |
| Alcohol use (n, %) | 195 (9.5) | 179 (9.7) | 16 (7.5) | 0.377 |
| Tobacco use (n, %) | 385 (18.7) | 347 (18.8) | 38 (17.9) | 0.835 |
| Anemia (n, %) | 812 (39.4) | 753 (40.7) | 59 (27.8) | <0.001 |
| CVD (n, %) | 871 (42.3) | 772 (41.8) | 99 (46.7) | 0.193 |
| CKD (n, %) | 618 (30.0) | 566 (30.6) | 52 (24.5) | 0.079 |
| Diabetes (n, %) | 842 (40.9) | 755 (40.9) | 87 (41.0) | 1 |
| Hyperlipemia (n, %) | 1412 (68.5) | 1298 (70.2) | 114 (53.8) | <0.001 |
| Hypertension (n, %) | 1378 (66.9) | 1246 (67.4) | 132 (62.3) | 0.151 |
| Respiratory (n, %) | 698 (33.9) | 554 (30.0) | 144 (67.9) | <0.001 |
| Malignancy (n, %) | 225 (10.9) | 197 (10.7) | 28 (13.2) | 0.312 |
| SBP (mmHg) | 133.0 (116.0- 152.0) | 133.0 (117.0- 152.0) | 133.0 (113.0- 154.3) | 0.577 |
| DBP (mmHg) | 72.0 (61.0- 85.0) | 72.0 (61.0- 85.0) | 71.0 (59.8- 86.0) | 0.711 |
| AST (IU/L) | 25.0 (19.0- 37.3) | 24.0 (19.0- 36.0) | 30.0 (21.0- 48.8) | <0.001 |
| ALT (IU/L) | 21.0 (14.0- 32.3) | 21.0 (14.0- 32.0) | 22.5 (15.0- 41.0) | 0.054 |
| Glucose (mmol/L) | 6.47 (5.4- 8.6) | 6.44 (5.4- 8.4) | 7.0 (5.6- 10.1) | 0.001 |
| Hemoglobin (mmol/L) | 12.9 (11.5- 14.2) | 13.0 (11.5- 14.2) | 12.3 (10.7- 13.7) | <0.001 |
| WBC (10^9^/L) | 8.6 (6.6- 11.6) | 8.6 (6.6- 11.4) | 9.6 (7.0- 14.4) | 0.001 |
| Lymphocyte (10^9^/L) | 1.4 (0.9- 2.00) | 1.4 (1.0- 2.0) | 1.2 (0.7- 1.7) | <0.001 |
| Platelet (10^9^/L)) | 228.0 (180.0- 284.0) | 229.0 (181.8- 284.0) | 224.5 (158.8- 281.8) | 0.116 |
| Potassium (mmol/L) | 4.2 (3.9- 4.6) | 4.2 (3.9- 4.6) | 4.2 (3.9- 4.6) | 0.973 |
| Sodium (mmol/L) | 139.0 (137.0- 142.0) | 139.0 (137.0- 142.0) | 138.0 (136.0- 141.3) | 0.06 |
| Temperature (°C) | 36.8 (36.6- 37.1) | 36.8 (36.6- 37.1) | 36.8 (36.5- 37.2) | 0.857 |
| HR (beats/min) | 82 (72- 96) | 82 (72- 95) | 89 (76- 105) | <0.001 |
| PTT (s) | 28.7 (25.8- 32.1) | 28.7 (25.9- 32.0) | 28.9 (25.6- 33.1) | 0.631 |
| RR (beats/min) | 18 (16- 22) | 18 (15- 22) | 21 (17- 25) | <0.001 |
| INR | 1.1 (1.0- 1.2) | 1.1 (1.0- 1.2) | 1.1 (1.0- 1.3) | <0.001 |
| GCS | 15.0 (14.0- 15.0) | 15.0 (14.0- 15.0) | 15.0 (13.0- 15.0) | 0.005 |
| SAPSII | 33.0 (26.0- 42.0) | 32.0 (25.0- 40.0) | 43.0 (35.8- 53.0) | <0.001 |
| OASIS | 30.0 (24.0- 36.0) | 29.0 (24.0- 35.0) | 37.0 (31.8- 43.0) | <0.001 |
| APSIII | 37.0 (28.0- 50.0) | 36.0 (27.0- 48.0) | 52.0 (38.8- 63.3) | <0.001 |
| Hospital length of stay (day) | 7.8 (4.4- 14.7) | 7.7 (4.5- 14.6) | 8.3 (4.2- 16.5) | 0.677 |
| ICU length of stay (day) | 2.7 (1.5- 5.7) | 2.5(1.4- 5.3) | 4.4 (2.4- 9.2) | <0.001 |

Abbreviation: GLR, glucose-to-lymphocyte ratio; CVD, cardiovascular disease; CKD, chronic kidney disease; SBP, systolic blood pressure; DBP, diastolic blood pressure; HR, heart rate; RR, respiratory rate; AST, aspartate aminotransferase; ALP, alkaline phosphatase; ALT, alanine aminotransferase; WBC, white blood cell count; INR, international normalized ratio; PTT, partial thromboplastin time; GCS, Glasgow coma scale; SAPSII, simplified acute physiology score II; OASIS, oxford acute severity of illness score; APSIII, acute physiology score III.

Figure S1. Decision curve analysis for prediction models of in-hospital mortality. (A) Decision curves showing the performance of APSIII, OASIS, and SAPSII with and without GLR in non-traumatic ICH patients; (B) Decision curves showing the performance of APSIII, OASIS, and SAPSII with and without GLR in ischemic stroke patients.


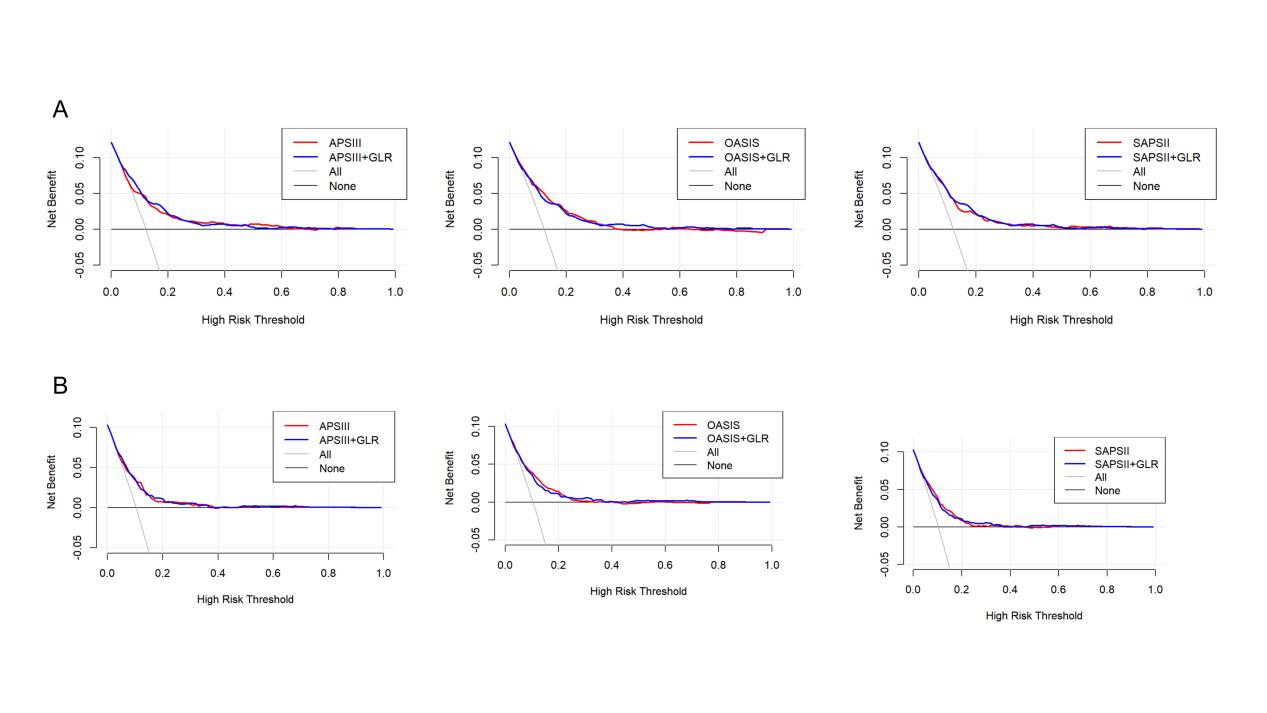

Supplement: Supplementary file 1 — Additional file 1. [file 40001_2025_3378_MOESM1_ESM.docx]
